# The Dermis as a Delivery Site of *Trypanosoma brucei* for Tsetse Flies

**DOI:** 10.1371/journal.ppat.1005744

**Published:** 2016-07-21

**Authors:** Guy Caljon, Nick Van Reet, Carl De Trez, Marjorie Vermeersch, David Pérez-Morga, Jan Van Den Abbeele

**Affiliations:** 1 Unit of Veterinary Protozoology, Department of Biomedical Sciences, Institute of Tropical Medicine Antwerp (ITM), Antwerp, Belgium; 2 Laboratory for Microbiology, Parasitology and Hygiene (LMPH), University of Antwerp, Wilrijk, Belgium; 3 Laboratory of Myeloid Cell Immunology, VIB Inflammation Research Center, Ghent, Belgium; 4 Unit of Parasite Diagnostics, Department of Biomedical Sciences, Institute of Tropical Medicine Antwerp (ITM), Antwerp, Belgium; 5 Unit of Cellular and Molecular Immunology, Vrije Universiteit Brussel (VUB), Brussels, Belgium; 6 Structural Biology Research Center (SBRC), VIB, Brussels, Belgium; 7 Center for Microscopy and Molecular Imaging (CMMI), Université Libre de Bruxelles (ULB), Gosselies, Belgium; 8 Laboratory of Molecular Parasitology, Université Libre de Bruxelles (ULB), Gosselies, Belgium; Faulty of Medicine, University of Calgary, CANADA

## Abstract

Tsetse flies are the sole vectors of *Trypanosoma brucei* parasites that cause sleeping sickness. Our knowledge on the early interface between the infective metacyclic forms and the mammalian host skin is currently highly limited. *Glossina morsitans* flies infected with fluorescently tagged *T*. *brucei* parasites were used in this study to initiate natural infections in mice. Metacyclic trypanosomes were found to be highly infectious through the intradermal route in sharp contrast with blood stream form trypanosomes. Parasite emigration from the dermal inoculation site resulted in detectable parasite levels in the draining lymph nodes within 18 hours and in the peripheral blood within 42 h. A subset of parasites remained and actively proliferated in the dermis. By initiating mixed infections with differentially labeled parasites, dermal parasites were unequivocally shown to arise from the initial inoculum and not from a re-invasion from the blood circulation. Scanning electron microscopy demonstrated intricate interactions of these skin-residing parasites with adipocytes in the connective tissue, entanglement by reticular fibers of the periadipocytic baskets and embedment between collagen bundles. Experimental transmission experiments combined with molecular parasite detection in blood fed flies provided evidence that dermal trypanosomes can be acquired from the inoculation site immediately after the initial transmission. High resolution thermographic imaging also revealed that intradermal parasite expansion induces elevated skin surface temperatures. Collectively, the dermis represents a delivery site of the highly infective metacyclic trypanosomes from which the host is systemically colonized and where a proliferative subpopulation remains that is physically constrained by intricate interactions with adipocytes and collagen fibrous structures.

## Author Summary

Sleeping sickness is caused by trypanosomes that are transmitted by the blood feeding tsetse flies. The present study has established an experimental transmission model with fluorescently labeled parasites in mice that allows us to study their fate following natural transmission by a tsetse fly bite. Parasites that arise in the tsetse salivary glands were found to be highly infective following inoculation in the mammalian skin in contrast with previous observations made for trypanosomes purified from the blood stream. This study unveiled that a proportion of parasites is retained in the skin and actively proliferates close to the initial inoculation site resulting in significantly elevated skin temperatures. This retention was linked to interaction with fat cells and collagen fibrous structures. Experimental transmission experiments were able to demonstrate that parasites can be acquired from the inoculation site immediately after the initial transmission.

## Introduction

Human African trypanosomiasis, also known as sleeping sickness, is indigenous for the African continent and is caused by two subspecies of *Trypanosoma brucei*, namely *T*. *brucei rhodesiense* and *T*. *brucei gambiense*. A range of other trypanosome species including *T*. *congolense*, *T*. *vivax* and *T*. *b*. *brucei*, is responsible for the majority of infections in livestock and wild animals. The current decline in number of sleeping sickness cases holds promise of moving into the eradication phase for this disease [1,2] whereas for animal trypanosomiasis with many problems of reservoir management and drug resistance [3–5] this goal is not yet in sight.

In tsetse flies, *T*. *brucei* parasites go through a complex developmental cycle in the alimentary tract and salivary glands ending with the cellular differentiation into the metacyclic form that can infect a new mammalian host [6,7]. Once this trypanosome population has been established in the salivary glands, it is maintained throughout the entire life span of the tsetse fly. The presence of high densities of trypanosomes in the salivary glands was shown to dramatically affect the tsetse saliva protein composition with a severe reduction of the major anti-hemostatic activities resulting in a hampered blood feeding process [8]. These infection-induced changes in the feeding physiology are believed to favor increased vector/host contact and enhance the chance of parasite transmission to a range of hosts. Only few studies have so far addressed the early parasitological features of a naturally transmitted trypanosome infection in the mammalian host. It is known that variant surface glycoproteins (VSGs) play a crucial role in the escape from immune elimination by the host adaptive immune response. Various glycoproteins [e.g. procyclin and BARP (*brucei* alanine-rich protein)] are displayed on the *T*. *brucei* surface in the tsetse fly vector [9] and VSGs are for the first time expressed at the metacyclic stage in the tsetse fly salivary glands. A range of metacyclic VSG antigen types (M-VATs) are present on the coats of the trypanosome population in tsetse saliva, which is considered beneficial for an infection establishment in mammals. Together with M-VAT expression, a set of predominant bloodstream VATs appears early during infection in the lymph and in the blood [10,11]. Experimental infections with purified bloodstream form (BSF) *T*. *brucei* and *T*. *congolense* revealed the stringent nature of the intradermal route that compromises the ability of these parasites to achieve host infection [12]. Tumor necrosis factor α (TNF-α) and inducible nitric oxide synthase (iNOS) were shown to contribute to the innate resistance to intradermal infection with low numbers of bloodstream form *T*. *congolense* parasites [12]. Addition of saliva to the inoculation mixture was also documented to enhance parasitemia onset following intrapinna BSF *T*. *brucei* injection, linked to a local immunosuppressive effect of saliva in the murine dermis [13]. Recently, a peptide with immunosuppressive properties has been identified from *G*. *morsitans* saliva with the ability to inhibit LPS induced MAPK signaling in splenocytes [14]. Together, these observations clearly indicate that the natural intradermal inoculation of metacyclic trypanosomes by tsetse flies adds a significant layer of complexity as compared to needle injection of bloodstream form trypanosomes in the peritoneal cavity used routinely in experimental infection models [15].

In humans, bites from infected tsetse flies regularly result in the formation of a skin ulceration or chancre [16]. This skin ulceration has also been documented during early *T*. *b*. *rhodesiense* infections in vervet monkeys [17] and following *T*. *congolense* infections in rabbits, goats, calves and sheep [18–21]. Histological and electron microscopy studies have documented the presence of parasites in the skin reactions [20,22–24]. Fluorescent trypanosomes or bioluminescent trypanosome versions have been used following needle injection to study tissue tropism and responses to drug treatment in the mammalian host [25–27] and to illustrate parasite genetic exchange in the tsetse fly vector [28]. In this study we have established tsetse mediated intradermal infections (i.e. a natural infection model) using fluorescently tagged trypanosomes to assess the kinetics of mammalian host colonization through the intradermal route using a combination of flow cytometry and molecular parasite quantification. This study unequivocally describes that metacyclic trypanosomes are highly infective and that a subpopulation of parasites is retained and actively proliferates in the dermis in close proximity of the initial inoculation site. This parasite retention was studied by scanning electron microscopy documenting a number of intricate interactions between parasites, adipocytes with characteristic periadipocyte collagenic baskets [29] and collagen bundles. These intradermal features and the induced temperature changes are compatible with the re-acquisition of parasites by the tsetse fly from this site. This study is the first to document in detail the presence of a residing intradermal trypanosome population at the tsetse biting site from which the host is systemically infected. These parasites may play a role as an early trypanosome reservoir in the mammalian host and could be picked up by the tsetse vector, demonstrated here for flies that fed soon after the primary inoculation.

## Materials and Methods

### Ethics statement

Mouse care and experimental procedures were performed under approval of the Animal Ethical Committee of the Institute of Tropical Medicine (Ethical clearance nr. VPU2014-1) and of the Vrije Universiteit Brussel (Ethical clearance nr. 13-220-1 and 15-220-14). Tsetse fly maintenance and experimental work was approved by the Scientific Institute Public Health department Biosafety and Biotechnology (SBB 219.2007/1410). The experiments, maintenance and care of animals complied with the guidelines of the European Convention for the Protection of Vertebrate Animals used for Experimental and other Scientific Purposes (CETS n° 123).

### Animals and parasites

C57BL/6JRj mice (Janvier) were used as animal model for natural trypanosome infections transmitted by tsetse flies. Tsetse flies (*Glossina morsitans morsitans*) were available at the Institute of Tropical Medicine, Antwerp. The flies originated from puparia collected in Handeni (Tanzania) and Kariba (Zimbabwe). The tsetse fly colony is maintained on an *in vitro* bovine blood feeding system at 26°C and a relative humidity of 65%-75%.

Three *Trypanosoma brucei* stocks were used for infection of tsetse flies and mice, the AnTAR1 strain and two transgenic lines of the pleiomorphic AnTat1.1E. *T*.*b*.*brucei* AnTAR1 is a post-tsetse fly strain derived from the EATRO 1125 stabilate that was originally isolated from a bushbuck in Uganda in 1966 and from which the AnTat1.1E strain was derived [30]. Two different transgenes were integrated in the tubulin array of AnTat1.1E using the constitutive trypanosomal expression vector pHD309: the genes encoding red fluorescent protein DsRed of a coral of the *Discosoma* genus (DsRED, Clontech) and a mutant of the *Aequorea macrodactyla* GFP-like protein (TagGFP2, Evrogen). Transfection and selection with 10 μg/ml hygromycine were performed as described in [25]. Epifluorescence microscopy was used to select the most fluorescent clones. The *in vitro* growth curves of the wild-type and AnTat1.1E^DsRED^ or AnTat1.1E^TagGFP2^ stocks were generated by seeding cells at 1 x 10^4^ cells ml^−1^ in 500 μl of HMI-9 with 15% fetal bovine serum in three replicate wells that were counted every 24 h.

### Establishment of *T*.*b*.*brucei* infections in tsetse flies

Teneral tsetse flies were fed 24–48 hours after emergence with a *T*.*b*.*brucei* AnTAR1, AnTat1.1E^DsRED^ or AnTat1.1E^TagGFP2^ infected blood meal supplemented with 10 mM reduced L-glutathione to increase trypanosome infection establishment [31]. Parasitized blood was harvested with heparin from cyclophosphamide-immune suppressed mice (Endoxan, Baxter) at 6–7 days post-infection and mixed with defibrinated horse blood (E&O Laboratories Limited) to obtain > 10^6^ BSF trypanosomes/ml in the initial blood meal. After this trypanosome-infected blood meal, flies were fed every 2–3 days on uninfected defibrinated horse blood. Salivary gland infected flies were selected by induced probing on pre-warmed glass slides that were microscopically examined for the presence of metacyclic trypanosomes. Flies with a mature, metacyclic salivary gland infection (SG+) were selected for infecting mice. Some SG+ flies were dissected to isolate the salivary glands and to evaluate by flow cytometry the spontaneous parasitic outflow from non-disrupted glands in phosphate saline glucose buffer (PSG; PBS pH 7.4 supplemented with 1% glucose).

### Establishment of natural *T*.*b*.*brucei* infections in mice through the tsetse fly bite

Mice were anesthetized by ketamine (100 mg/kg) and xylazine (20 mg/kg) and infected with *T*.*b*.*brucei* AnTAR1, AnTat1.1E^DsRED^ or AnTat1.1E^TagGFP2^ parasites by allowing individual infected tsetse flies (1 fly for each mouse) to probe on the mouse ear pinnae. In order to assess the parasitemia development in the peripheral blood, 1:200 diluted tail vein blood samples were analysed using improved Neubauer counting chambers and Uriglass disposable slides (Menarini Diagnostics) for parasitema levels < 10^7^/mL. When using fluorescently tagged trypanosomes, parasite appearance in the bloodstream was also monitored by flow cytometry using the FACSVerse and BD FACSsuite software.

At regular time points after infection (4.5, 18, 42, 66 and 90 hpi), mice were euthanized and blood, cervical and mandibular lymph nodes, and ears were collected. Tissues were either preserved in RNA*later* according to the manufacturer’s recommendations for RT-qPCR, fixed in 2.5% EM-grade glutaraldehyde 0.1M cacodylate pH7.2 for scanning electron microscopy or collected in phenol red-free RPMI 1640 medium (Life Technologies) for further processing for flow cytometry analysis. Blood samples collected by a cardiac puncture were subjected to erythrocyte lysis (1:10 addition of 8% ammonium chloride, 0.84% sodium bicarbonate 1mM of EDTA pH 7.3). Lysis was conducted for 10 minutes on ice followed by centrifugation at 870 × *g* for 8 minutes at 4°C. The pellet was rinsed with RPMI and centrifuged again at 870 × *g* at 4°C for 8 minutes. The pellet was resuspended in RNA*later*, kept at 4°C overnight and, after removal of RNA*later*, snap-frozen in liquid nitrogen and stored at -80°C. Harvested ears and cervical and mandibular lymph nodes were incubated overnight in RNA*later* at 4°C, snap-frozen and stored at -80°C.

### Experimental dose-controlled *T*.*b*.*brucei* infections in mice

Various doses of purified bloodstream form (BSF) and metacyclic form (MCF) trypanosomes were used for experimental infection experiments using an intradermal injection method. BSF parasites were expanded in C57Bl/6JRj mice and purified from their heparinized blood, using diethylaminoethyl cellulose (DEAE52) anion exchange chromatography. Parasites were collected in PSG buffer (PBS pH 7.4 supplemented with 1% glucose), centrifuged at 870 × *g* and resuspended just prior to injection in sterile LPS-free PBS and kept on ice. MCF parasites were collected as the outflow from SG+ glands, washed to remove tsetse saliva components and resuspended in PBS. To exclude differences as a result of the anion exchange procedure, MCF parasites were also subjected to an anion exchange chromatography in one experiment. Motile/viable parasites were counted using the Uriglass disposable slides and live parasite concentration adjusted in order to initiate experimental trypanosome infections intradermally with various parasite doses. In one experiment, viability of DEAE52 purified MCF and BSF parasite was verified by flow cytometry using 7AAD (Via-Probe, BD). The inoculation consisted of a single injection of 20 μl buffer containing 500, 200, 100, 50, 20 or 5 BSF or MCF trypanosomes between the ventral and dorsal ear dermal layer using a 30 gauge insulin microsyringe.

### Thermographic recording and thermal data analysis

High resolution thermographic imaging of mice exposed to a *T*.*b*.*brucei* AnTAR1 infective tsetse bite (on the left mouse ear pinnae) was achieved using the Testo 890 thermal camera equipped with a 42° lens. Acquisition was at a 640 × 480 resolution with a thermal sensitivity (NETD) of 40 mK and an instantaneous field of view (IFOV) of 0.11 mm. The emissivity was fixed at 0.95. The reflected temperature (RTC) was set to 20°C. Images were analysed using the IRsoft software provided by the camera manufacturer. Surface temperatures of dorsum and ear extremities were recorded to evaluate the effect of infection on the total body temperature and to assess the impact of dermal (left ear) parasite presence on local skin temperature.

### Tsetse fly feeding and parasite acquisition

The potential effect of parasite-induced thermal changes in the skin on tsetse feeding was simulated by an *in vitro* feeding system. Tsetse flies were fed in obscurity on defibrinated horse blood through a silicone membrane equilibrated at either 25.1°C or 24.0°C corresponding to the measured average surface temperatures of respectively a heavily parasitized ear dermal site and the contralateral control site. The effect of this thermal difference was also evaluated at higher temperatures, i.e. by comparing the feeding response at 29.0°C and 30.2°C.

In order to evaluate the availability of skin-residing trypanosomes for acquisition by tsetse flies, a total of 6 mice in two independent experiments were exposed on the venter to the bites of 7 *T*.*b*.*brucei* AnTAR1 SG+ infected flies. This primary exposure results in the deposition of a skin-residing parasite population. Eighteen hours after this primary exposure, mice were challenged with a new set of flies on the venter (*n* = 15/mouse) and the dorsum (opposite to primary exposure, *n* = 15/mouse). For each mouse and exposure side, 10 flies that engorged a full blood meal were dissected 1 hour after the blood feeding and abdomens were collected in 50 μl PSG. Tissues were next homogenized in Trizol reagent (Invitrogen) and stored at -80°C until further processing. In order to evaluate that the 18 hpi dermal parasites can establish infections in tsetse flies, sets of 40 teneral male tsetse flies were allowed to feed on dorsal and ventral sides. Seven days later, tsetse flies were dissected to microscopically determine the midgut infection status.

### Flow cytometry and fluorescence microscopy

Blood samples for flow cytometry analysis were obtained from the tail vein and diluted 1/100 in RPMI 1640 without phenol red (Life Technologies) supplemented with penicillin (10U/μl), streptomycin (10U/μl), L-glutamine, 10 μg/ml anti-Fc antibody and 25 U/ml heparin. Dissected lymph nodes and ears were placed in a petri dish with 2 ml RPMI 1640 without phenol red (Life Technologies) and with penicillin, streptomycin and L-glutamine, 100μg/ml Liberase TL (Roche) and 50μg/ml DNase (Sigma) similar to the procedure described elsewhere [32]. The ears were separated in ventral and dorsal dermal sheets. The different tissues were crushed using a syringe plunger and the samples were incubated at 37°C, ears for one hour and lymph nodes for thirty minutes to maximally liberate cells from these tissues. After incubation, ice cold PBS supplemented with 2mM EDTA and 10% of fetal calf serum was added to stop the enzymatic digestion. This solution with the tissue debris was filtered through a 70 μm cell strainer (BD) and cells were collected by centrifugation at 870 × *g* for 8 minutes at 4°C. Cells were resuspended in phenol red-free RPMI 1640 with 10 μg/ml anti-Fc antibody and 25 U/ml heparin and stained with 7-amino-actinomycin D (7AAD, BD Via-Probe Cell Viability Solution) and anti-mouse CD45 APC-Cy7 (clone 30-F11, eBioscience) for exclusion of potentially autofluorescent white blood cells. At least 2x10^5^ events were acquired for each sample in a volumetric analysis on the FACSVerse flow cytometer (BD) and analysed with BD FACSSuite Software. To identify *T*.*b*.*brucei* AnTat1.1E^DsRED^ and AnTat1.1E^TagGFP2^ parasites, a parasite gate was set in the FSC/SSC density plot based on the cell characteristics of an *in vitro* trypanosome culture. CD45+ and 7AAD+ (dead) cells were excluded.

Parasites were also visualized in saliva deposits on glass slides by conventional light and epifluorescence microscopy. Confocal microscopy in mounted ear dermal sheets was performed by using the Zeiss LSM700 microscope equipped with the ECPlan-Neofluar 40×/1.30 Oil DIC M27 objective lens. Videos of 3 s were obtained by combining 10 consecutive images made in a 14.1 s time window.

### Dermal sections and scanning electron microscopy

Glutaraldehyde-fixed ear samples were embedded in 1% agarose and cut into 500 μm sections with a Vibratome (Leica). Sections were then incubated overnight at 4°C in 2.5% glutaraldehyde, 0.1 M cacodylate buffer (pH 7.2), and post-fixed in 2% OsO4 in the same buffer. After serial dehydration, samples were dried at critical point and coated with platinum by standard procedures. Observations were made in a Tecnai FEG ESEM QUANTA 200 (FEI) and images processed by the SIS iTEM software (Olympus).

### RNA extraction

RNA*later*-treated samples stored at -80°C were thawed and weighted. Ears (separated in ventral and dorsal dermal sheets) and lymph node tissue samples were transferred into lysing matrix D homogenization vessels (MP Biomedicals LLC), containing Lysis/Binding Solution (10–12 μL/mg tissue) of the RNAqueous Kit (Ambion) followed by homogenization with FastPrep (MP Biomedicals LLC). This lysing matrix was identified as the most appropriate based on a pilot experiment that compared lysing matrixes A, D, F and S. Ear samples were homogenized twice at 6.0 m/s for 40 seconds and lymph node samples once. After homogenization, the lysates were centrifuged at top speed (21130 × *g*) for 2 minutes in order to remove tissue debris that may be present in the lysate. RNA isolation was carried out with the RNAqueous Kit (Ambion, Life technologies) according to the manufacturer’s recommendations. Potential DNA contamination was enzymatically removed with 2U RNAse-free DNase I (Ambion) followed by inactivation using the DNase inactivation reagent (Ambion) and heat treatment at 75°C for 10 minutes.

RNA from the tsetse fly abdomens was extracted using the Trizol reagent followed by 2 consecutive chloroform extractions, RNA precipitation in the presence of Glycoblue and two 75% ethanol washes of the RNA pellet. RNA was resuspended in 20 μl DEPC water (Ambion). Samples were subjected to DNAse I treatment (Biolabs) for 10 minutes at 37°C. The reaction was stopped by the addition of 5 mM EDTA and heat inactivation for 10 minutes at 75°C. The concentration and purity of the RNA extracts was measured by using a NanoDrop spectrophotometer (Isogen, Life Science ND-1000). Absorbance ratios at 260/280nm and 260/230nm were determined as measures for RNA-purity. Samples were stored at -80°C until further use.

### Quantitative RT-PCR

Parasite presence in the various tissues was quantified using the spliced-leader (SL) RNA specific RT-qPCR as described elsewhere [33]. Briefly, RNA was reverse transcribed and cDNA amplified using the SensiFAST SYBR No-ROX One-Step Kit (Bioline) in a reaction volume of 20 μl with 1x SensiFast containing 400 nM of reverse and forward primers (5’-CAATATAGTACAGAAACTG-3’ and 5’-AACTAACGCTATTATTAGAA-3’), 0.2 μL Reverse Transcriptase and 0.4 μL RNase inhibitor. Thermal cycling conditions were 10 minutes incubation at 45°C, 2 minutes at 95°C, followed by 40 cycles of 95°C for 5 seconds, 50°C for 10 seconds, 60°C for 5 seconds. Post-amplification melting curves were recorded from 45°C to 95°C, with increments of 0.1°C and continuous acquisition. Cp values were obtained from the amplification curves by the second derivative approach and Tm calling was used to confirm the presence of a single amplicon.

### Graphs and statistical analysis

Flow cytometry analyses were performed in a volumetric mode, allowing for the accurate calculation of the parasite concentrations in the sample and the actual number of recovered parasites from the tissues using the BD FACSSuite Software. RT-qPCR based calculation of parasite presence in the tissues was based on linear regression of Cp values obtained for a standard curve (containing 2×10^4^, 10^4^, 5×10^3^, 2.5×10^3^, 10^3^, 5×10^2^, 10^2^, 10, 1 parasites). *In vitro* parasite doubling times (T_d_) and median infectious parasite doses were calculated by using non-linear regression fitted with an exponential equation in GraphPad Prism 6.0. The same software was used for preparing the graphs and for the statistical analysis (two-tailed unpaired Mann-Whitney-*t* test, one-way ANOVA) of the data. Data were represented as means ± standard error of the mean. *P* values ≤ 0.05 were considered to be statistically significant.

## Results

### Establishment of a tsetse fly infection model with fluorescently tagged *T*. *brucei*

**Fig 1:**
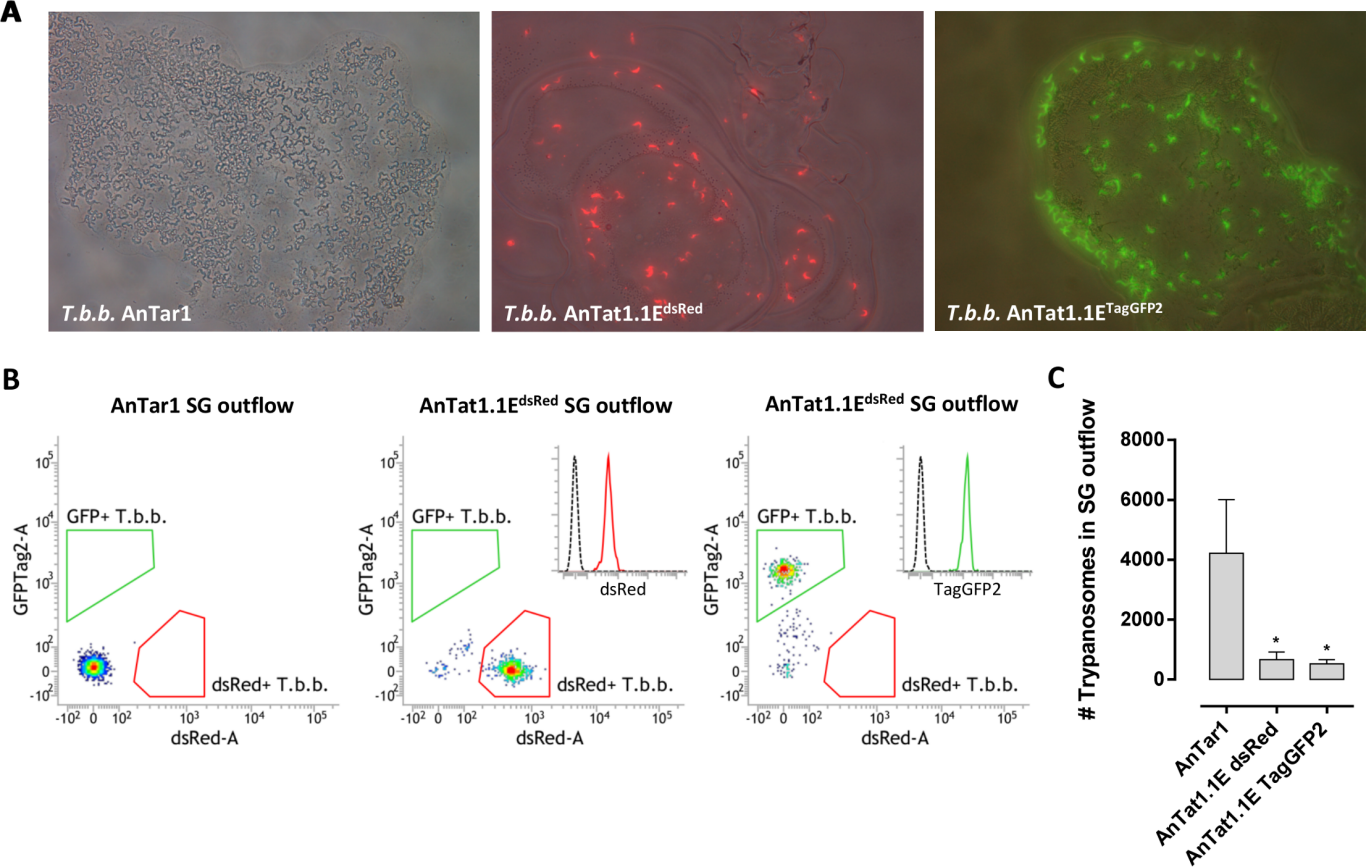
Infection establishment of different *T*.*b*.*brucei* strains in *G*.*m*.*morsitans* tsetse flies. (**A**) Tsetse saliva deposits containing metacyclic trypanosomes of the *T*.*b*.*brucei* AnTAR1, AnTat1.1E^dsRed^ or AnTat1.1E^TagGFP2^ strains. Microphotographs are merged brightfield and fluorescence images of saliva deposited on pre-warmed glass slides by SG+ flies infected with the respective parasite strains. (**B**) Flow cytometric analysis of salivary gland outflows containing AnTAR1, AnTat1.1E^dsRed^ or AnTat1.1E^TagGFP2^ metacyclic trypanosomes. Fluorescent populations are indicated in the appropriate gates. Histogram insets illustrate the dsRed and TagGFP2 fluorescence in the AnTat1.1E transgenic metacyclics in comparison with the non-tagged AnTAR1 population (dotted line). (**C**) Trypanosome numbers in the outflows of non-disrupted salivary gland pairs from individual flies (*n* = 5 /group) infected with one of the three different parasite strains. These differences in parasite density in the salivary glands between the wildtype and the reporter strains were also clear by microscopical inspection in eight independent experiments for the comparison *T*.*b*.*brucei* AnTAR1 (total *n* = 1666 flies) vs. AnTat1.1E^dsRed^ (*n* = 2091) and two independent experiments for the comparison *T*.*b*.*brucei* AnTAR1 (*n* = 245) vs. AnTat1.1E^TagGFP2^ (*n* = 228). Parasite numbers in the salivary glands were analysed by flow cytometry. * *P* < 0.05

Lines of *T*.*b*.*brucei* AnTat1.1E transfected to express the *dsred* or *taggfp2* transgene were used to infect *G*.*m*.*morsitans* tsetse flies. These two fluorescent strains displayed the same *in vitro* doubling times as the wildtype (respectively 7.0 ± 0.5 h and 7.5 ± 0.2 h versus 6.9 ± 0.5 h). In comparison with the *T*.*b*.*brucei* AnTAR1 parental strain, lower salivary gland infection rates were recorded in flies infected with the *T*.*b*.*brucei* AnTat1.1E^DsRED^ and AnTat1.1E^TagGFP2^ strains (S1 Table). This was linked to a lower maturation index of the transgenic parasites in the fly given that midgut infection rates following feeding on a reduced L-glutathione supplemented parasitized blood meal exceeded 95% for all tested strains. Following salivary gland colonization (SG+), it was observed that parasite densities in the saliva deposits on pre-warmed glass slides were higher in the *T*.*b*.*brucei* AnTAR1 as compared to the fluorescently tagged *T*.*b*.*brucei* AnTat1.1E-infected flies (Fig 1A). Parasite outflows from individually dissected salivary gland pairs were volumetrically analysed by flow cytometry (Fig 1B) and shown to contain about 8-fold less metacyclic parasites (*p* < 0.05) as compared to AnTAR1 SG+ flies (Fig 1C). Fluorescently tagged trypanosomes were used for analysing parasite kinetics from the infection initiation site following an infective tsetse fly bite.

### Kinetics of *T*. *brucei* following a tsetse fly mediated intradermal inoculation

**Fig 2:**
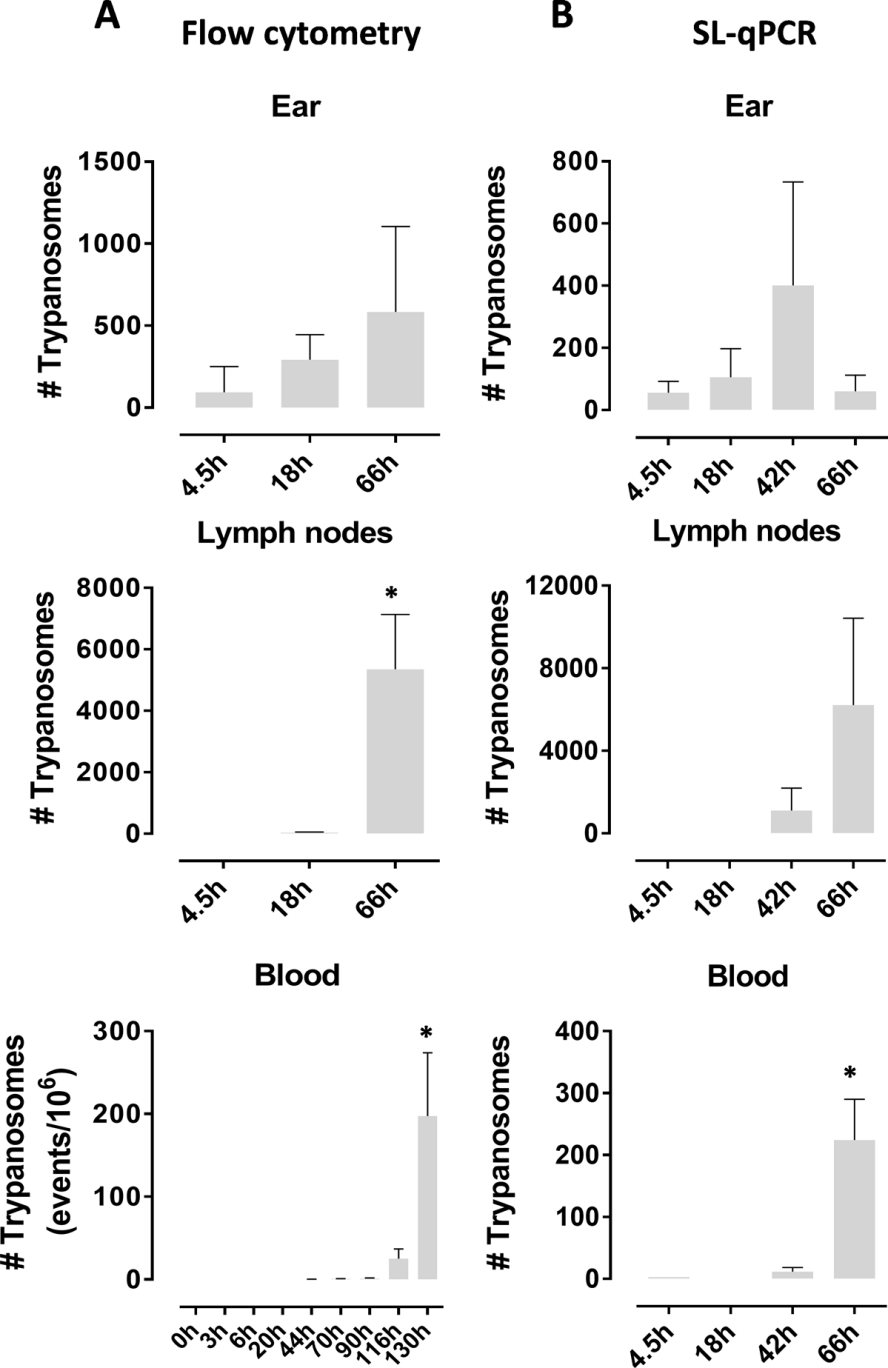
Host tissue colonization by the parasite following a tsetse mediated transmission. Numbers of parasites in the ears, draining lymph nodes and peripheral blood of mice exposed to SG+ tsetse fly bites as determined by volumetric flow cytometry detecting the dsRed transgene (**A**) and by SL-RNA specific RT-qPCR (**B**). Data shown are from three individual mice for each time point. Each ear was exposed to the bites of an individual infected tsetse fly. No statistical significant differences were recorded between the two different quantification methods. These parasite kinetics data are representative of two independent experiments. Data are shown as the mean ± SEM. *: *P* <0.05

**Fig 3:**
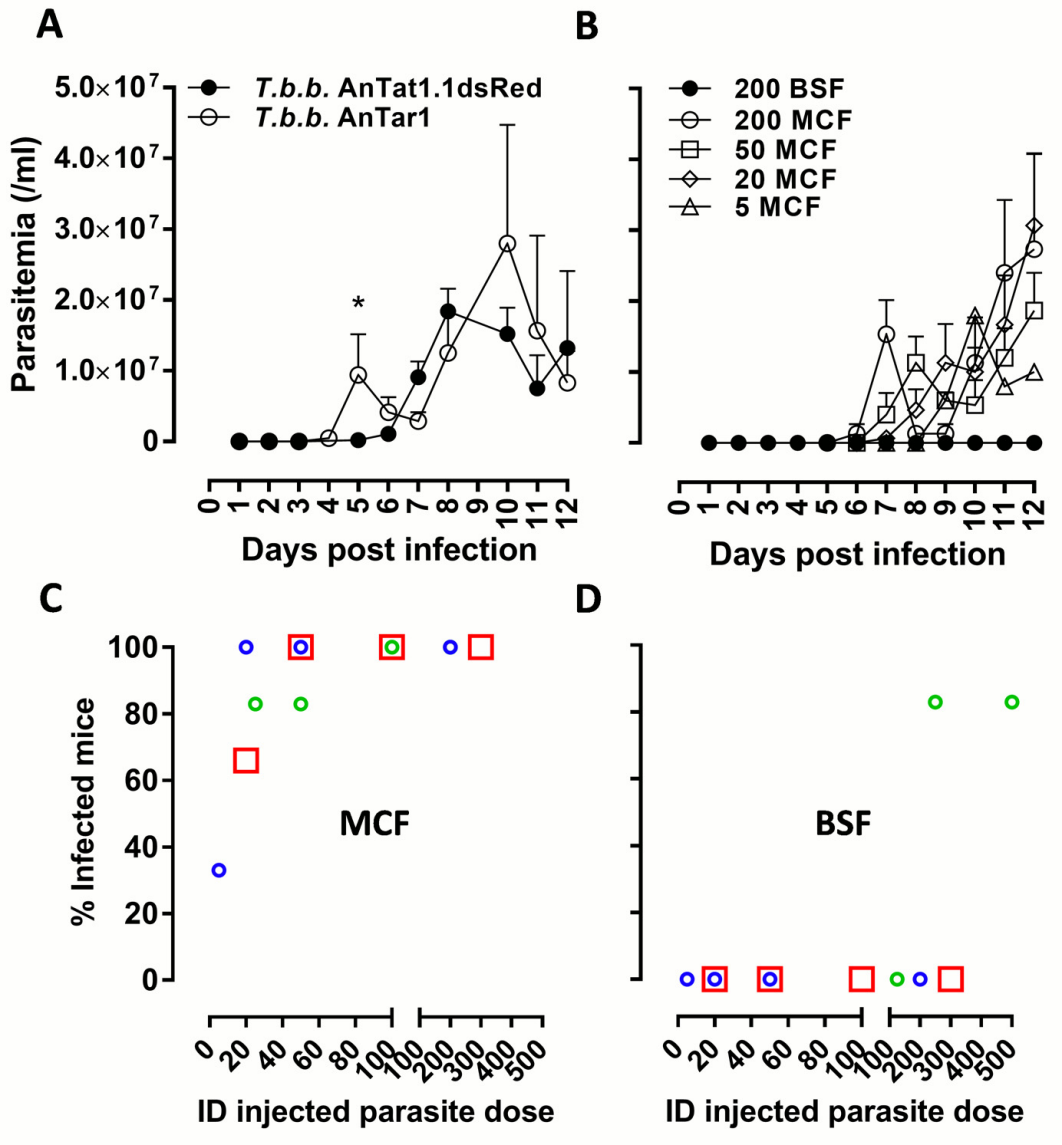
Effect of trypanosome dose on intradermal infection. (**A**) Parasitemia development in peripheral blood of mice (*n* = 5/group) naturally infected by the bites of *T*.*b*.*brucei* AnTAR1 (empty symbols) and *T*.*b*.*brucei* AnTat1.1EdsRed infected tsetse flies (filled symbols) with respectively a high and low number of parasites in the salivary glands. (**B**) Parasitemia progression in peripheral blood of mice (*n* = 3/group) intradermally inoculated with varying numbers of metacyclic parasites (200, 50, 20 and 5 MCF) extracted from SG+ *G*.*m*.*morsitans* tsetse flies versus 200 purified bloodstream (BSF) *T*.*b*.*brucei* parasites. These data are representative of 3 independent experiments. Infection rates are recorded following intradermal needle inoculation of varying numbers of (**C**) washed (round symbols) or DEAE52-purified (red squares) metacyclic (MCF) *T*.*b*.*brucei* parasites isolated from tsetse salivary glands (*n* = 3 or 6 for each parasite dose and experiment, totaling 48 mice) and (**D**) DEAE52-purified bloodstream (BSF) *T*.*b*.*brucei* parasites (*n* = 3 or 6 for each parasite dose and experiment, totaling 42 mice). The parasitological status was checked for 14 days following parasite inoculation. Different symbol colours indicate the 3 independent infection experiments. Different sizes of the symbols are to avoid overlaps. Red squares correspond to the BSF infection rates calculated from the infection experiment that also included the DEAE52-purified MCF parasites as shown in panel C. Data are shown as the mean ± SEM.

In order to simulate a natural transmission in a mouse model, *T*.*b*.*brucei* AnTat1.1E^DsRED^ infected *G*.*m*.*morsitans* flies were used to initiate intradermal trypanosome infections in mouse ear pinna followed by analysis of host colonization by the parasites. Early kinetics of the emigration of fluorescently labeled parasites from the dermal inoculation site to the lymphoid tissue and blood stream were examined by flow cytometry and confocal microscopy. Fluorescently tagged *Trypanosoma* parasites were detected in the ear dermis at the various time points analysed in this setup (4.5, 18 and 66hpi; Fig 2A). Parasite presence was also quantified by an SL-RNA specific RT-qPCR (Fig 2B) confirming the presence and expansion of parasites in the ear dermis during the early course of infection (Fig 2B). Analysis of the kinetics of trypanosome emigration revealed that parasites could be detected by 18hpi in the lymph nodes draining the dermal site of infection (Fig 2A). Then, around 42–44 hpi, parasites were detected in the peripheral blood using flow cytometry and qPCR (Fig 2A and 2B). In order to compare parasitemia onset following natural inoculation of different parasite doses, infected flies with a high or a low parasite density in the saliva (i.e. infected with either the *T*.*b*.*brucei* AnTAR1 or the AnTat1.1E^dsRED^ strain) were used to initiate intradermal infections in the ears of mice followed by conventional parasitological detection in the peripheral blood. This suggested that infection with a lower parasite dose resulted in a delayed appearance of parasites in the blood circulation, whereas peak parasitemia levels were not significantly altered (Fig 3A). Excluding that this observation results from intrinsic differences between the wildtype and dsRed transgenic strain, experimental needle inoculation of various doses of the *T*.*b*.*brucei* AnTAR1 metacyclic parasites confirmed this inverse correlation between inoculated parasite dose and parasitemia onset in the peripheral blood (Fig 3B). In addition, metacyclic (MCF) trypanosomes showed to be highly capable of host colonization through this intradermal route in contrast to purified bloodstream forms (BSF, Fig 3C versus 3D). Indeed, for the intradermal needle injection of metacyclic parasites, 7 parasites was calculated as the 50% infectious dose with doses as little as 5 metacyclic trypanosomes often resulting in no establishment of infection under the used experimental conditions. Purification of metacyclic trypanosomes by anion exchange chromatography was found not to reduce the infectivity (Fig 3C, red squares). A dose as high as 200 bloodstream form parasites was not infective through the same intradermal route. A cell viability assay was performed on the prepared DEAE52-purified BSF and MCF inoculums, revealing very comparable viability (≥ 95%) just prior to injection. Assessment of post-injection viability approximately 3 hours after preparing the samples revealed a slightly elevated cell death in the BSF (16%) as compared to the MCF sample (5%) (S2 Fig). Some variation was observed in the independent BSF infection experiments (Fig 3D), but it can be stated that the critical threshold for infection of mice with DEAE52-purified *T*.*b*.*brucei* AnTAR1 is situated around 200–300 BSF parasites in contrast to a > 10-fold lower critical threshold observed for the MCF parasites.

### Intradermal persistence of *T*. *brucei* following an infected tsetse fly bite

**Fig 4:**
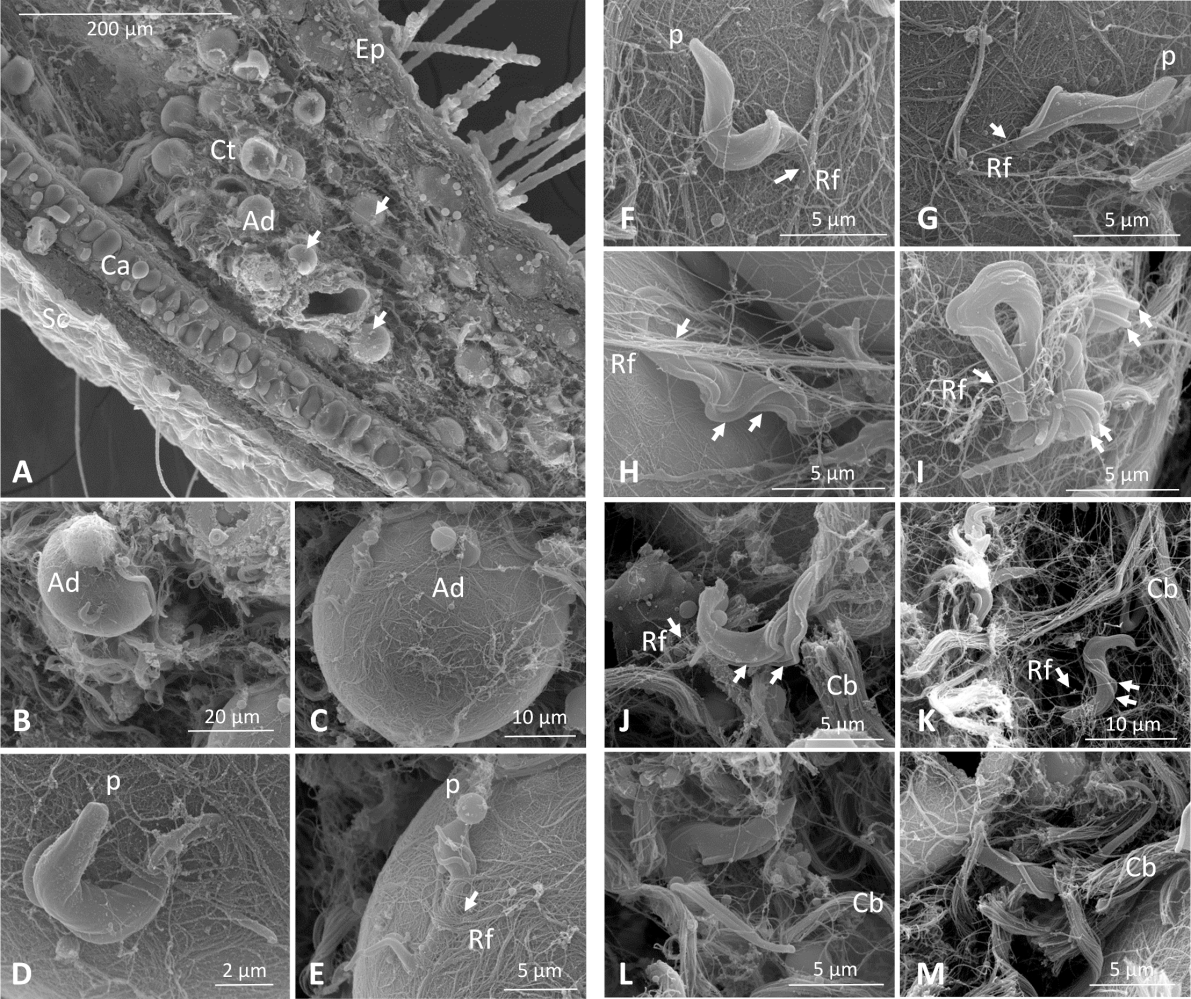
Parasite retention and entanglement at the dermal infection initiation site. Scanning electron microscopy images of 90 hpi dermal ear sections illustrating presence of parasites in the connective tissue close to the cartilage layer of the ear. Subcutaneous adipocytes were readily identified by the smooth external appearance of their cytoplasmic membrane and the characteristic presence of a surrounding collagen network known as basket [34]. Parasites are indicated with white arrows (**A**). Intricate interactions of trypanosomes with adipocytes in the connective tissue were observed frequently (**B**-**G**). Parasites were found with the anterior part buried inside the adipocyte (**D**-**G**). Parasites were prominently entangled by reticular fibers (white arrows) of the periadipocytic baskets (**E- H**) and are embedded in collagen bundles (**J**-**M**). Despite these interactions, trypanosomes were proliferating as many cells had multiple flagella (**H**-**J**). These observations were made in two independent scanning electron microscopy experiments with a total of eight parasite infected ears at 90 hpi. Sc, stratum corneum; Ep, epidermis; Ad, adipocyte; Ct, connective tissue; Ca, cartilage; Rf, reticular fibers; p, posterior trypanosome end; Cb, collagen bundle.

**Fig 5:**
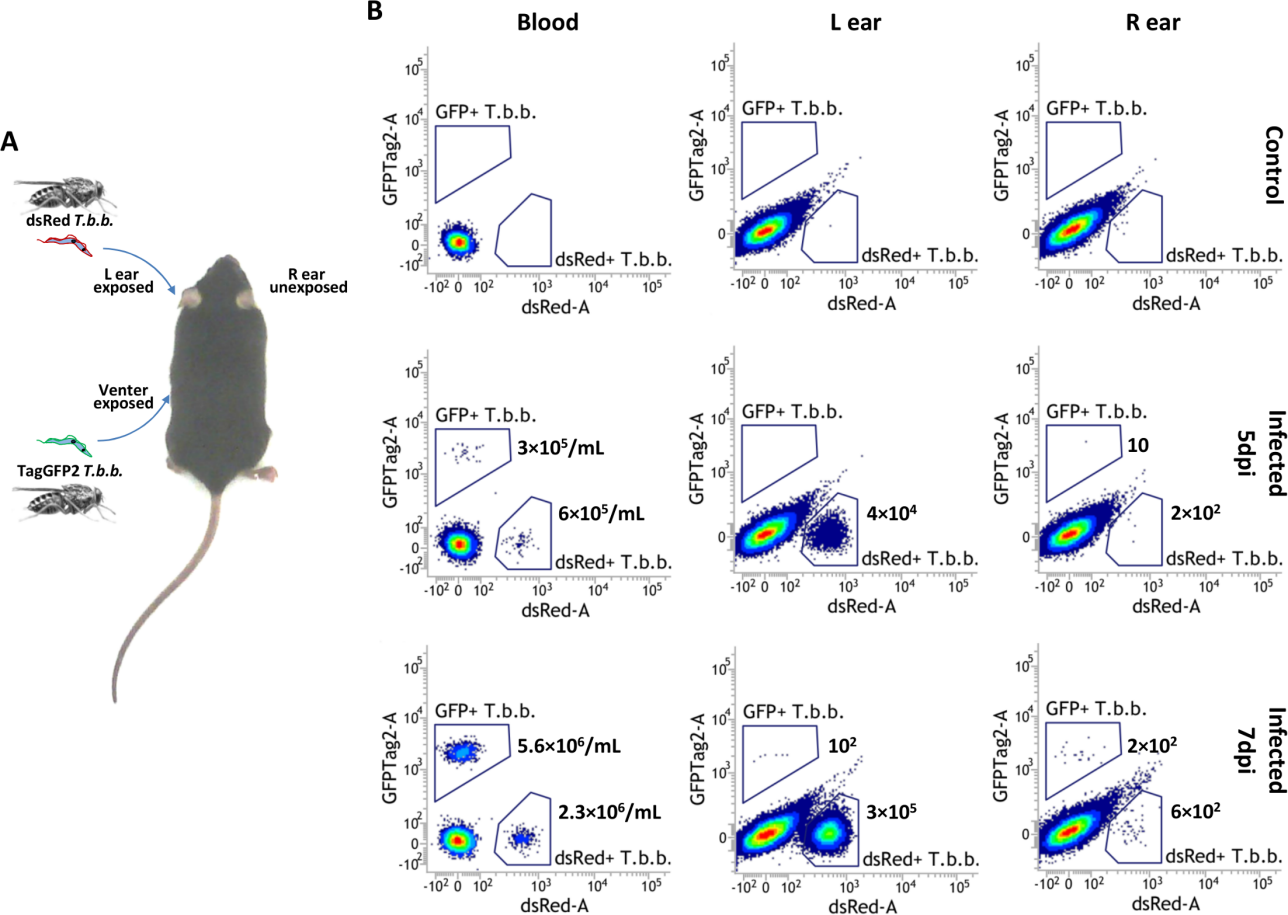
Parasite persistence and expansion at the dermal site of infection. (**A**) Experimental setup using natural infection with two differentially labeled parasite strains (AnTat1.1E^dsRed^ or AnTat1.1E^TagGFP2^) to determine the origin of the parasites expanding at the dermal inoculation site. Mice were exposed to the bites of infected tsetse flies to introduce AnTat1.1E^dsRed^ parasites in the left ear and AnTat1.1E^TagGFP2^ at the abdominal side. (**B**) Flow cytometry profiles at 5 and 7 dpi illustrating the presence of the two parasite strains in the peripheral blood and localized expansion of only the dsRed strain in the ear dermis exposed to the infected tsetse fly bite. Shown data are the CD45^-^ events within the trypanosome gate. Parasite concentrations in the blood and total number of parasites in the tissue extracts are indicated. This experiment was with two mice and one control mouse for each time point with a total of six mice.

Intradermal inoculation of *T*.*b*.*brucei* AnTat1.1E^DsRED^ or AnTat1.1E^TagGFP2^ by the bites of SG+ tsetse flies resulted in a trypanosome population residing inside the dermis (left ear) whereas this population could not be detected at the contralateral side of the mouse body (right ear) (S1 Fig). Scanning electron microscopic analysis of vibratome ear sections revealed the entanglement of skin residing parasites through different apparent mechanisms including (i) intricate interactions with adipocytes that are present in the connective tissue in close proximity to the cartilage layer of the ear (Fig 4A–4G) (ii) entanglement by reticular fibers (Rf) of the periadipocytic baskets (Fig 4E–4I) and those in the interstitial spaces (Fig 4J–4K) and (iii) embedding in collagen bundles (Cb, Fig 4J–4M). Interaction with the adipocytes mainly involved burying of the anterior part of the parasites into the fat cells, leaving the flagellar pocket accessible for nutrient uptake (Fig 4D–4G). Consistent with these various modes of entanglement, trypanosomes were characterized by a restricted anterior-posterior movement inside the dermis as evidenced by a confocal microscopy analysis inside mounted ear dermal sheets (S1 Video). Despite these structural interaction features, numerous parasites were observed to be proliferative as indicated by the presence of a double flagellum (Fig 4H–4K). Trypanosomes with a double flagellum were also observed on the surface of adipocytes which strongly suggests parasite multiplication (Fig 4H–4I). Moreover, evaluation of the viability by 7-amino-actinomycin D (7AAD) staining demonstrated very limited numbers (< 1%) of non-viable trypanosomes during the expansion phase in the dermis indicating that the observed interactions do not have a detrimental impact on the parasite. The absence of significant numbers of non-viable parasites in the dermal trypanosome population is also demonstrated in the confocal microscopy video (S1 Video). In order to evaluate whether the expanding parasite population is derived from the initial inoculum or from parasites of the peripheral blood that re-colonized the dermal site, mice were naturally infected with two differentially labelled parasites (respectively the dsRed and TagGFP2 expressing *T*.*b*.*brucei* AnTat1.1E) on remote sites of the mouse body (left ear pinna and venter, Fig 5A). This infection resulted in a mixed trypanosome population in the peripheral blood (Fig 5B) whereas the expanding population reaching > 10^5^ at the ear inoculation site expresses a single fluorescent marker (dsRed) corresponding to the initial inoculum. No expanding parasite population could be detected at the contralateral side of the body (right ear pinna). The dermal parasite population seems to reduce to low numbers of remaining dsRed-expressing *T*.*b*.*brucei* AnTat1.1E detected by flow cytometry at 18 dpi despite parasite presence in the peripheral blood (S3 Fig).

### Thermal changes in the dermis and acquisition of trypanosomes by tsetse flies

**Fig 6:**
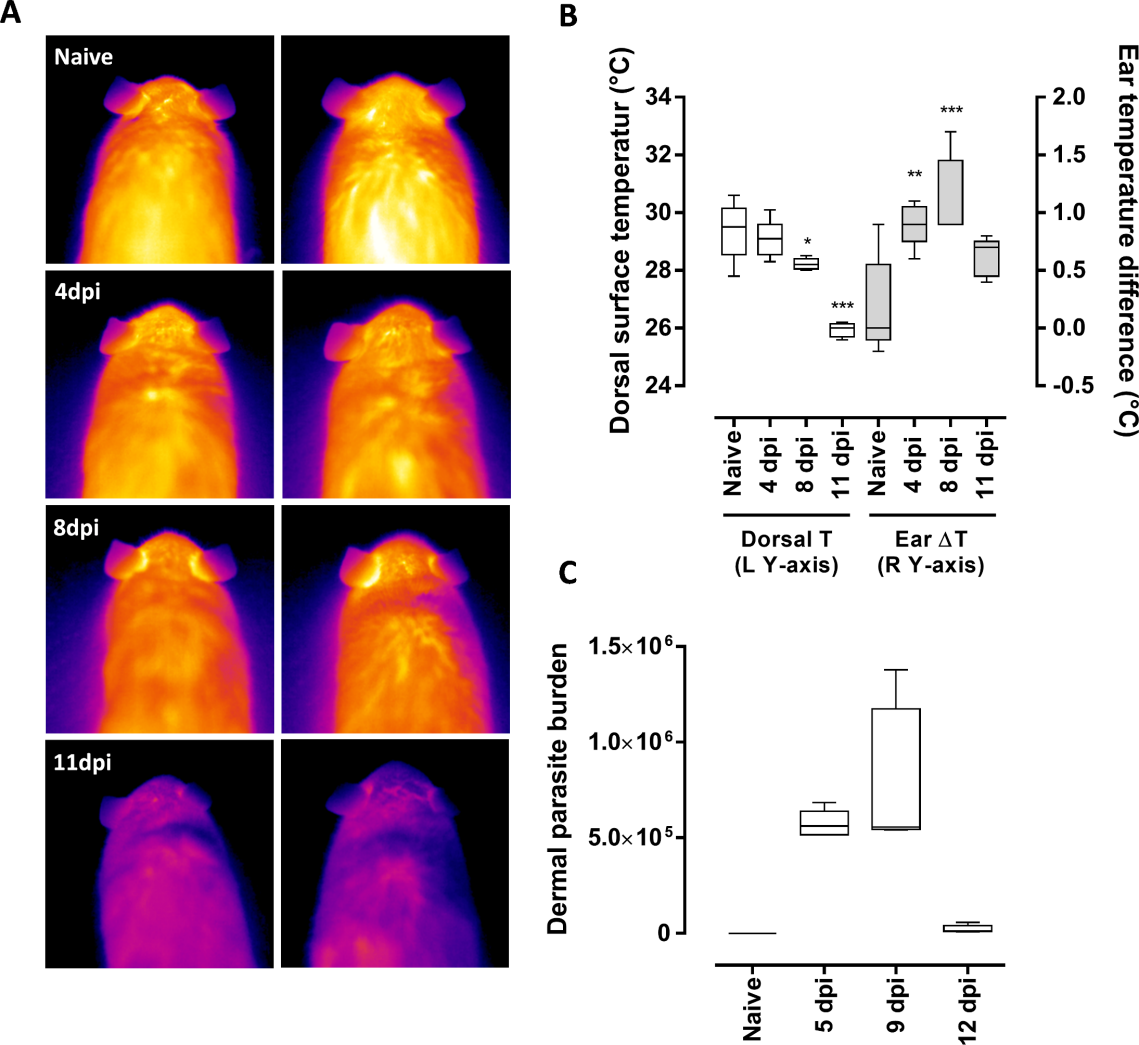
Thermographic analysis of the dermal site of parasite expansion. (**A**) High resolution thermographic images of 2 representative naive mice and mice at 4, 8 and 11dpi following initiation of a *T*.*b*.*brucei* AnTAR1 infection by an SG+ tsetse fly bite on the left ears (*n* = 5 for each time point). Right ears were not exposed and served as controls. Images are presented with identical thermal scales. Data are representative of two independent experiments (**B**) Box plot graph with the measured dorsal temperatures (plotted on left Y-axis) and the temperature differences between exposed and non-exposed ears (plotted on the right Y-axis; *n* = 5 for each group and time point). (**C**) Evolution of the density of parasites in the dermis of the ear determined by SL-RNA specific RT-qPCR of the mice used for thermographic imaging (*n* = 5 for each group and time point). Box plots are with whiskers extending from minimum to maximum. *: *P* < 0.05, **: *P* < 0.01; ***: *P* < 0.001

Following exposure of the left ear pinna to SG+ tsetse fly bites, thermal changes in the infected mouse were measured at various time points (4, 8 and 11 days post-infection) with an emphasis on the differences between the temperatures of the inoculated left ears and those of the non-exposed right ears (Fig 6A and 6B). An overall lethargy with significant hypothermia was observed from 8 dpi with a more than 3°C reduction in recorded surface body temperatures by 11 dpi as compared to non-infected littermates (*p* < 0.0001). Parasite exposed ear pinnae were characterized by a significantly increased temperature (25.1 ± 0.1°C) relative to the non-exposed ear (24.0 ± 0.2°C) with an average elevation of 1.1 ± 0.2°C at 8 dpi (*p* < 0.0001; Fig 6B). This difference in temperature over the course of infection closely correlated with the ear dermal parasite burden as determined by SL-RNA specific RT-qPCR in the same mice used for the thermographic analyses (Fig 6C). To evaluate whether the thermal difference could potentially be of any physiological importance for the tsetse fly feeding responses, 3-day starved flies were offered an artificial horse blood meal through a silicone membrane with surface temperatures representing the mean left and right ear temperatures measured at 8 dpi (25.1°C versus 24.0°C respectively). Significantly more flies (59.1%, *n* = 176) obtained a blood meal from the 25.1°C surface compared to only 39.9% (*n* = 176) at 24.0°C (*p* = 0.0003, S2 Table). The tsetse engorgement rate was not significantly altered upon temperature increase from 29.0°C (84.3%, *n* = 70) to 30.2°C (79.7%, *n* = 68). This suggests that the increased temperature induced by the dermis-residing trypanosome infection could represent an attractive cue for tsetse fly feeding in a specific, low temperature range.

**Fig 7:**
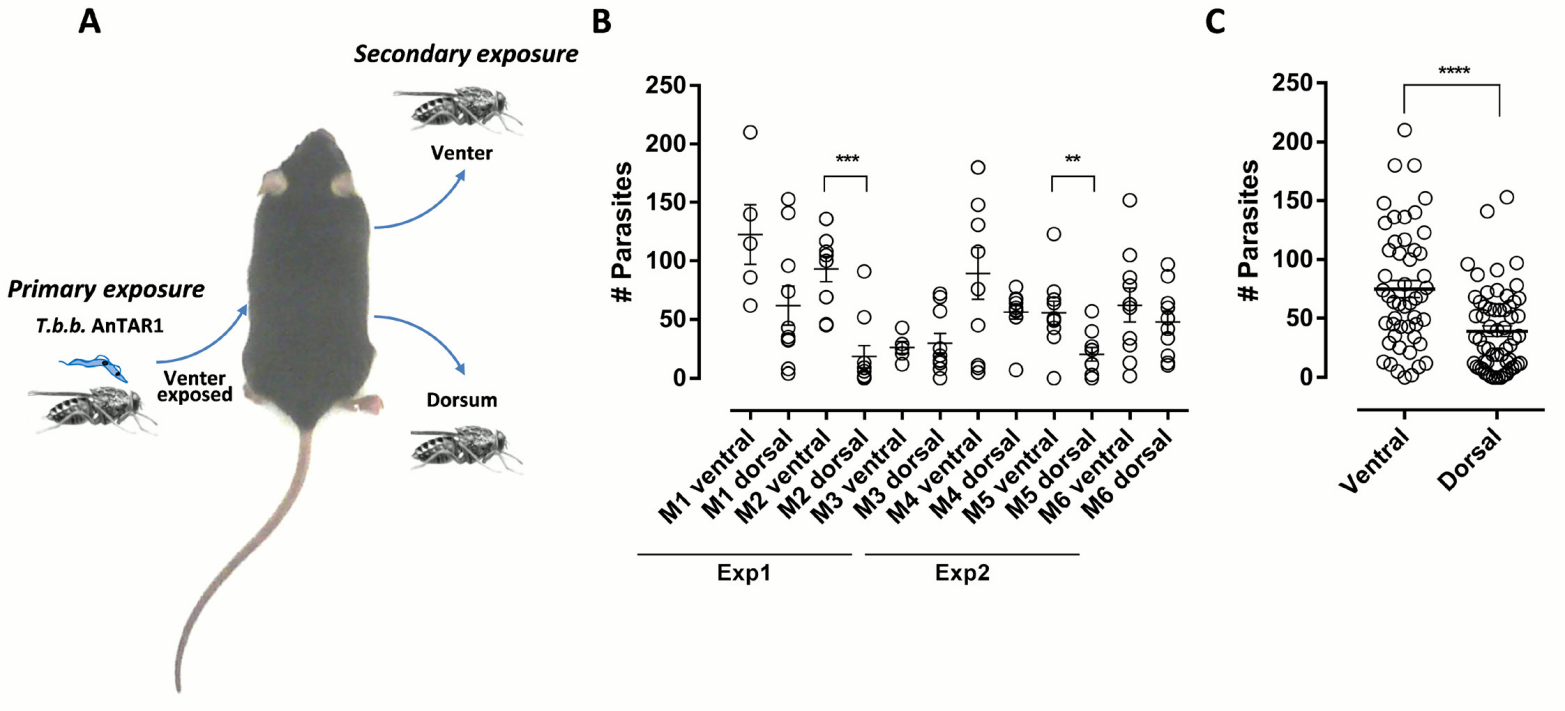
Parasite acquisition by tsetse flies from the infected dermal site. (**A**) Experimental setup indicating the initial infection of mice by exposure of the mouse dorsum to *T*.*b*.*brucei* AnTAR1 SG+ tsetse fly bites, followed 18 hpi by feeding of teneral tsetse flies on the venter or dorsum. (**B**) Parasites acquired during the blood feeding process were quantified by SL-RNA specific RT-qPCR on individual tsetse fly abdominal extracts (*n* = 5–10 fully engorged flies fed on an individual side and mouse). Data for the individual mice (M1-6) from two independent experiments with number of parasites acquired from dorsal and ventral sides. (**C**) Combined data of parasite numbers acquired by individual flies from the ventral (*n* = 50) and dorsal sides (*n* = 60). **: *P* < 0.01; ***: *P* < 0.001; ****: *P* < 0.0001

In order to assess whether this skin residing parasite population can be acquired by these feeding tsetse flies, freshly emerged tsetse flies were fed on the ventral and dorsal side of mice that were prior exposed at the ventral side to the bites of multiple *T*.*b*.*brucei* SG+ tsetse flies, 18 hours earlier (Fig 7A). After feeding, ingested parasites were quantified through SL-RNA RT-qPCR on RNA extracted from the abdomens of flies that engorged a full blood meal. On average, higher parasite numbers were recovered from tsetse flies that were fed on the ventral side which corresponds to the primary *T*.*b*.*brucei* SG+ exposure site (75 parasites versus 39 from the dorsal side, *p* < 0.0001; Fig 7B and 7C). Parasite acquisition by tsetse from the non-exposed dorsal side indicated that exposure to the multiple tsetse fly bites at the ventral side already resulted in parasite presence in the peripheral blood within 18h after exposure. In two independent experiments using a total of 160 flies, blood fed flies were evaluated for the establishment of a midgut trypanosome infection by dissection and microscopic evaluation at 7 days post feeding. We could not detect the establishment of infections in the tsetse fly midgut following uptake of parasite populations from the dorsal or ventral side at this early time point of infection.

## Discussion

A significant body of research on African trypanosomes is conducted by making use of needle injection of bloodstream parasites in the peritoneal cavity of the murine host. Although many breakthroughs in understanding the biology and immunology of trypanosomes have been made through such experimental models [15], very few information is yet available on the parasitological features of naturally transmitted trypanosome infections. In this study we have used *T*. *b*. *brucei* AnTAR1, AnTat1.1E^DsRED^ and AnTat1.1E^TagGFP2^ for natural transmission studies using the savannah tsetse fly, *Glossina morsitans* that represents a proficient vector for *T*. *brucei*. Although these parasites display the same *in vitro* replication times, the two fluorescent transgenic strains showed lower metacyclic infection frequencies and lower parasite densities in the salivary glands. This may be explained by a non-neutral characteristic of the *dsred* and *taggfp2* transgenes or effects related to the integration site (tubulin array) resulting in a less virulent phenotype in the fly. Exploiting these differences between the dsRed-tagged AnTat1.1E and AnTAR1 strain in terms of numbers of parasites in the tsetse salivary glands, this study was able to compare the early parasitemia progression following a naturally inoculated low and an average 8-fold higher parasite dose, respectively. Combination with experimental needle injections of varying doses of the same parasite strain (AnTAR1), to exclude intrinsic strain differences in the host colonization process, revealed that parasite dose largely determines the time of appearance in the peripheral blood, without affecting peak parasitemia levels. A study by Wei *et al* has shown that the intradermal route of *T*. *brucei* and *T*. *congolense* parasite transmission differs considerably from the experimental peritoneal route and constitutes a stringent bottleneck for infection establishment [12]. These observations were made following intradermal inoculation of purified blood stream form trypanosomes. In this study, these experimental conditions were replicated for the infectious metacyclic trypanosomes purified from the tsetse fly salivary glands, demonstrating that the natural intradermal transmission route does not impose a significant constraint for infection establishment by these salivary gland derived forms. The 50% infectious doses were calculated to be 7 metacyclic (MCF) trypanosomes whereas even 200 bloodstream (BSF) trypanosomes were unable to establish an infection upon intrapinna injection in mice. This clearly suggests that the metacyclic forms of *T*.*brucei* are far better prepared than bloodstream forms to survive and develop at the intradermal inoculation site in the mammalian host. The infective dose of metacyclic trypanosomes in mice that we determined in our study is > 50-fold lower than the one reported for a *T*.*brucei rhodesiense* strain in humans. In this human infection study, it was concluded that an average man would require a subcutaneous dose of 300–450 metacyclic parasites to develop a systemic infection. However, a high variability was observed with a lowest infective dose of 170 and the highest non-infective dose of 1067 parasites [35]. All these experimental observations suggest that the MCF infective dose delivered through tsetse intradermal inoculation is variable and host/parasite-dependent.

Metacyclic parasites are known to be in a quiescent non-proliferative stage. Recent comparisons between MCF trypanosomes in the tsetse salivary gland and BSF trypanosomes indicated some transcriptional differences between these different life cycle stages including metabolic genes (glycolysis, phosphorus metabolism), surface molecules, transporters (ion, glucose, amino acid and nucleoside transporters), transcriptional regulators and translation machinery components [36] but it is not clear what could be the basis for the higher infectivity of metacyclic trypanosomes. The higher infectivity of metacyclic trypanosomes did not relate to the previously described immunomodulatory activity of soluble salivary factors [13] given that parasites were washed prior to inoculation. As TNF and iNOS were found implicated in determining the dermal infection bottleneck for *T*. *congolense* [12], susceptibility of metacyclic *T*. *brucei* parasites to locally produced levels of these effectors would be of interest for further exploration. Interestingly, an ultrastructural study suggested some specific characteristics of the intracutaneous forms of *T*. *brucei* with smaller mitochondria and rough endoplasmatic reticulum as compared to the MCF and BSF trypanosomes [20] suggesting a specific trypanosome subpopulation (stage) at this site. This is strengthened by our clear observations that a parasite subpopulation remained and proliferated in proximity of the initial inoculation site following a tsetse-mediated infection. The presence of significant numbers of parasites has been previously documented in local skin reactions induced by various trypanosome species (*T*. *brucei* and *T*. *congolense*) [20]. Tsetse fly mediated *T*. *b*. *rhodesiense* infections induced chancres in > 50% of vervet monkeys within 4 to 8 days post-infection [17]. In goats, the inoculation of a single metacyclic *T*. *brucei* parasite was reported to be sufficient for the typical ulceration [19]. Cannulation of the lymphatics in goats revealed that parasites can be detected in the lymph within 1–2 days after the infective tsetse fly bite with the formation of a chancre at the infection initiation site by the third day [10]. Our studies in the murine model confirm this rapid migration to the lymph, with detectable levels of parasites in the draining lymph nodes within 18 hours post-infection. Despite this rapid migration to the lymph, a proportion of trypanosomes was found to remain and proliferate in the mouse ear dermis where parasite levels larger than 10^5^ were detected by SL-RNA RT-qPCR and flow cytometry. Using mixed infections with differentially labeled trypanosomes, the expanding intradermal trypanosome population (expressing the dsRed transgene) was unequivocally shown not to result from a re-invasion from the bloodstream. The kinetics of differentiation of the inoculated metacyclic into blood stream trypanosomes in the skin remains to be further explored but will require the identification of suitable surface exposed discriminative markers. A transmission electron microscopy (TEM) study in goat skin chancres between 3 and 11 dpi demonstrated a constantly high percentage (50–75%) of aberrant trypanosome forms with strong vacuolization which were therefore considered to be degenerating and non-dividing forms [20]. Another study in sheep [24] reported the same cell death phenomenon for *T*. *congolense* in the dermis coinciding with the chancre formation. However, a study in New Zealand White rabbits only observed parasite degeneration by 11 dpi [22]. Our cell viability staining (7AAD-based) of fluorescently tagged intradermal *T*. *brucei* trypanosomes in naturally infected mice did not reveal significant cell death in the parasite population during the early progressive phase of parasite expansion as determined up to 7 dpi. These apparent host differences might correlate to the fact that beyond a certain degree of edema no chancres were found to develop in this mouse intrapinna model. RNA-based parasite quantification demonstrated an abrupt reduction in dermal parasite numbers by 12 dpi corresponding to the observations made in rabbits and was further confirmed by a strong reduction in the dermal parasite population as observed by flow cytometry at 18 dpi. Confocal microscopy analyses on separated ear sheets illustrated a clear motility of the bulk of parasites during the expansion phase (see S1 Video). Scanning electron microscopy (SEM) on ear tissue vibratome sections confirmed the proliferation of the dermal parasites as exemplified by the presence of double flagella. A previous study also described the occurrence of proliferative as well as “giant” forms of *T*. *brucei* with multiple nuclei and several axonemes per flagellum [20]. Interestingly, our SEM data additionally illustrate intricate interactions of the anterior end of trypanosomes with adipocytes. This interaction seems to be very tight as indicated by the twisting and folding of the flagellar membrane (Fig 4G). Intriguingly, the entanglement did not implicate the posterior end, leaving free the flagellar pocket, the site for endocytosis. Similar interactions have also been observed in SEM analyses of *T*. *cruzi* cocultures with *in vitro* cultured adipocytes [37]. Of importance, *T*. *cruzi* infection was shown to influence the secretion by adipocytes of insulin-response regulatory adipokines [37] and skin adipocytes also represent a source of antimicrobial peptides during bacterial infections [38]. Possibly, these interactions might provide an advantage to the parasite in terms of metabolic requirements or exposure to cellular or humoral components of the immune system. In view of the observed intricate interactions with *T*. *brucei* parasites, adipocytes remain an understudied cell type in the regulation of metabolism and immunity during African trypanosomiasis. In this context, recent findings demonstrated that adipose tissue is a functional parasite reservoir for *T*.*brucei* during systemic development in the mammalian host where parasite gene expression is remodeled to metabolically adapt to the utilization of lipids as a carbon source [39].

Collagen fibers of the periadipocyte baskets acted synergistically in supporting the interaction of trypanosomes with these skin adipocytes. Parasite cell bodies were discovered in several images to be heavily entangled by collagen microfibers while other parasites were found embedded between collagen bundles in the connective tissue. Although the presence of trypanosomes in close proximity to the collagen has been described in early histology studies [23] and TEM studies [22,24], the degree of entanglement observed in the present SEM images had not yet been fully appreciated. It is known that extracellular matrix proteins are used by pathogens for adhesion and invasion (reviewed in [40]). *T*. *brucei* was found to secrete an active prolyl oligopeptidase that cleaves collagen and thereby might regulate its interactions with collagen in the extracellular matrix [41]. The interactions with collagen and adipocytes could be responsible for retention of parasites in the dermis but could also play an important role in creating a physiologically or immunologically privileged site that is beneficial for the inoculated parasites allowing them to proliferate and establish a locally adapted subpopulation as suggested by our study. The observation that *in vitro* culture of *T*. *brucei* was more efficient in the presence of collagen-producing fibroblasts [42] is strengthening this hypothesis. In these cultures aberrant multinucleate and giant forms similar to those described inside the chancre [20] were also observed upon surpassing the maximal growth phase. The support of *T*. *b*. *gambiense* culture by primary murine bone marrow derived cells was found to require the indispensable presence of adipocyte clusters [43], which also points to a positive influence of adipocytes and collagen on parasite expansion.

High resolution thermographic imaging revealed significant changes in the skin surface temperatures correlating with the intradermal parasite burden. We have interrogated whether the recorded temperature increase could represent a thermal cue for tsetse flies in favour of parasite acquisition. Unexpectedly, nearly 60% of 3-day starved tsetse flies acquired a blood meal when the surface temperature of the artificial membrane was equilibrated at 25.1°C corresponding with the ear temperature at a high parasite burden. In contrast, less than 40% of flies blood fed at a temperature (24.0°C) corresponding to the non-inflamed ear tissue. Previous electrophysiological studies demonstrated that the firing frequency of thermosensitive cells on the tarsi of tsetse flies increased linearly in a temperature range from 24°C to 34°C which could underlie the observed difference in feeding response [44]. At a temperature around 30°C, around 80% of the flies acquired a blood meal, but no impact of a 1.2°C difference (between 29.0°C and 30.2°C) could be noted in our feeding experiments. At temperatures from 37°C up to 42°C, tsetse flies were documented to acquire blood meals more efficiently with shorter mean feeding times at elevated temperatures [45]. Collectively, the trypanosome carrying dermal site with an elevated local temperature will be a good location for tsetse fly blood feeding in a recently infected mammalian host. Indeed, once landed on the host skin, local temperature sensed by antennal and tarsal thermosensors will be an important determinant for tsetse probing activity [44,46]. Tsetse flies also seem to have preferential landing sites and feed at concentrated feeding sites, particularly on the lower front legs of cattle [47], which therefore represents a site prone to trypanosome deposition and acquisition. Our results indicate that parasite acquisition by tsetse flies can occur during the first time period from the primary exposure site where the infection was initiated although parasites at 18 hpi seemed not yet able to colonize the tsetse midgut. Nevertheless, parasites that are present in the skin could potentially play a role as a transient reservoir for early uptake by tsetse flies soon after their deposition into the skin of animals with low to undetectable parasite concentrations in the peripheral blood. This type of role has been recently described for *Leishmania infantum* parasites that persisted for several months in lesions at the primary inoculation sites in dogs and from where sand flies could efficiently acquire the parasite [48]. The contribution of the dermal trypanosome population in chronic, low parasitemic models as a reservoir for tsetse fly infection, for recrudescence and its susceptibility to drug treatment remain to be further established.

Collectively, this study illustrates the high intradermal infectivity of metacyclic trypanosomes and retention of a parasite subpopulation proximal to the initial inoculation site. The expanding dermal trypanosome population is engaged in a number of remarkable interactions with skin adipocytes and the extracellular matrix. In addition, parasites at this site induced changes in the skin surface temperature. Further unraveling of the basis of the skin-resident trypanosome phenotype will shed new light on processes underlying host colonization and parasite acquisition by the tsetse fly vector.

## Supporting Information

S1 Table Transmissibility of different *T*.*b*.*brucei* strains in *G*.*m*.*morsitans* tsetse flies. Teneral male tsetse flies were infected with *T*.*b*.*brucei* AnTAR1, AnTat1.1E^dsRed^ or AnTat1.1E^TagGFP2^ in the presence of 10 mM reduced L-glutathion. Parasite infection in the salivary gland was evaluated by induced probing on pre-warmed microscopy slides. Indicated are the frequencies within 4 weeks after infection of (i) flies with metacyclic trypanosomes in the saliva and therefore harboring a mature infection, (ii) flies with immature long forms but no metacyclic trypanosomes in the deposited salivary secretions and (iii) flies that did not develop a salivary gland infection as determined by a parasitologically negative probing result. The differences between the wildtype and transgene-expressing strains have been observed in eight independent experiments for the comparison *T*.*b*.*brucei* AnTAR1 (*n* = 1666 flies used in total for infection) vs. AnTat1.1E^dsRed^ (*n* = 2091) and two independent experiments for the comparison *T*.*b*.*brucei* AnTAR1 (*n* = 245) vs. AnTat1.1E^TagGFP2^ (*n* = 228).
(DOCX)

S2 Table Feeding response of flies in function of increasing temperature. Feeding response of 3-day starved flies on an artificial horse blood meal through a silicone membrane. Membrane surface temperatures were set to match the average left infected ear and the right uninfected ear temperatures measured at 8 dpi (25.1°C and 24.0°C respectively) and at a higher temperature (29.0°C and 30.2°C). Represented data are the percentages of flies fed within 5 minutes. (N) = total number of flies in the feeding experiment; * a two-tailed Chi-square test was performed.
(DOCX)

S1 Fig Parasite retention and proliferation at the dermal infection initiation site. Flow cytometry profiles illustrating the detection of (**A**) AnTat1.1E^dsRed^ or (**B**) AnTat1.1E^TagGFP2^ trypanosomes in the infection initiation site (L ear) and absence in the contralateral side (R ear). Shown events are all singlets in the ear dermal extracts at 90 hpi. These observations are representative of 2 independent experiments with two or three mice/group with a total of nine mice.
(TIFF)

S2 Fig Parasite viability in the inoculum. The viability of the DEAE52-purified MCF and BSF parasites was assessed by 7AAD-based staining and flow cytometry analysis in (**A**) the parasite inoculum and (**B**) the inoculum approximately 3h after preparation. Percentage viable trypanosomes are indicated in the dot plots. The parasites in this viability analysis correspond to the infection rates indicated by red squares in Fig 3C and 3D for the MCF and BSF parasites respectively.
(TIFF)

S3 Fig Parasite persistence at a late infection time point. Flow cytometry profiles of control mice and mice at 18 dpi following natural infection with two differentially labeled parasite strains (AnTat1.1E^dsRed^ or AnTat1.1E^TagGFP2^). Mice were exposed to the bites of infected tsetse flies to introduce AnTat1.1E^dsRed^ parasites in the left ear and AnTat1.1E^TagGFP2^ at the abdominal side. Dot plots illustrate the presence of the two parasite strains in the peripheral blood and low numbers of remaining AnTat1.1E^dsRed^ parasites in the ear dermis exposed to the infected tsetse fly bite. Shown data are the CD45^-^ events within the trypanosome gate. Parasite concentrations in the blood and total number of parasites in the tissue extracts are indicated. This experiment was with three mice and one control mouse.
(TIFF)

S1 Video Parasite movement inside the dermis. Movement of *T*.*b*.*brucei* AnTat1.1EdsRed parasites recorded by confocal microscopy inside the ear dermal sheets at 90 hpi. Parasites display a typical anterior-posterior movement consistent with a physical constraint inside the dermis.
(AVI)
