# Supplementary material for: The Dermis as a Delivery Site of Trypanosoma brucei for Tsetse Flies
Source: PLoS Pathog. 2016 Jul 21;12(7):e1005744. doi: 10.1371/journal.ppat.1005744 (PMC4956260; doi:10.1371/journal.ppat.1005744)
Supplement: S1 Table — Teneral male tsetse flies were infected with T.b.brucei AnTAR1, AnTat1.1EdsRed or AnTat1.1ETagGFP2 in the presence of 10 mM reduced L-glutathion. Parasite infection in the salivary gland was evaluated by induced probing on pre-warmed microscopy slides. Indicated are the frequencies within 4 weeks after infection of (i) flies with metacyclic trypanosomes in the saliva and therefore harboring a mature infection, (ii) flies with immature long forms but no metacyclic trypanosomes in the deposited salivary secretions and (iii) flies that did not develop a salivary gland infection as determined by a parasitologically negative probing result. The differences between the wildtype and transgene-expressing strains have been observed in eight independent experiments for the comparison T.b.brucei AnTAR1 (n = 1666 flies used in total for infection) vs. AnTat1.1EdsRed (n = 2091) and two independent experiments for the comparison T.b.brucei AnTAR1 (n = 245) vs. AnTat1.1ETagGFP2 (n = 228). (DOCX) [file ppat.1005744.s001.docx]

| **Probing result** | ***T.b.b.* AnTAR1** | | ***T.b.b.* AnTat1.1E^dsRed^** | | ***T.b.b.* AnTat1.1E^TagGFP2^** | |
| --- | --- | --- | --- | --- | --- | --- |
|  | **Total** | **Frequency (%)** | **Total** | **Frequency (%)** | **Total** | **Frequency (%)** |
| **Metacyclic** | 46 | 37 | 16 | 16 | 13 | 12 |
| **Immature long forms** | 9 | 7 | 15 | 15 | 24 | 23 |
| **Negative** | 70 | 56 | 70 | 69 | 69 | 65 |
| **Total Flies** | 125 | 100 | 101 | 100 | 106 | 100 |
