# Supplementary material for: The Dermis as a Delivery Site of Trypanosoma brucei for Tsetse Flies
Source: PLoS Pathog. 2016 Jul 21;12(7):e1005744. doi: 10.1371/journal.ppat.1005744 (PMC4956260; doi:10.1371/journal.ppat.1005744)
Supplement: S2 Table — Feeding response of 3-day starved flies on an artificial horse blood meal through a silicone membrane. Membrane surface temperatures were set to match the average left infected ear and the right uninfected ear temperatures measured at 8 dpi (25.1°C and 24.0°C respectively) and at a higher temperature (29.0°C and 30.2°C). Represented data are the percentages of flies fed within 5 minutes. (N) = total number of flies in the feeding experiment; * a two-tailed Chi-square test was performed. (DOCX) [file ppat.1005744.s002.docx]

|  | Surface t (°C) | Feeding response |  |
| --- | --- | --- | --- |
| **Exp. I** | 24.0 | 39.8 % (176) | p<0.0003 * |
|  | 25.1 | 59.1 % (176) |  |
|  |  |  |  |
| **Exp. II** | 29.0 | 84.2% (70) | p=0.48 |
|  | 30.2 | 79.7 % (69) |  |
